# Tumour SNPs Associated with Immune-Related Hepatitis in Patients with Melanoma Receiving Immune Checkpoint Inhibitors

**DOI:** 10.3390/biomedicines13102351

**Published:** 2025-09-25

**Authors:** Jose María Rodríguez-Piñas, Alicia Romero-Lorca, Maria Gaibar, Apolonia Novillo, Margarita Rubio, Diego Malón, Beatriz Antón-Pascual, Francisca Inmaculada Camacho, Mercedes Cavanagh, David Ricardo Luján, Ana Manuela Martín, Radia Khedaoui, Diana Moreno, Fernando Pinedo, Macarena Boiza, Silvia García-Adrián, Patricia Gómez, David Marrupe, Ana Fernández-Santander

**Affiliations:** 1 Department of Medicine, Faculty of Medicine, Health and Sports, Universidad Europea de Madrid, 28670 Madrid, Spain; josemaria.rodriguez@universidadeuropea.es (J.M.R.-P.); margarita.rubio@universidadeuropea.es (M.R.); franciscainma.camacho@ext.universidadeuropea.es (F.I.C.); m.cavanagh@salud.madrid.org (M.C.); 2 Human Genetic Variability Group, Hospital La Paz Institute for Health Research–IdiPAZ (La Paz University Hospital–Universidad Autónoma de Madrid–Getafe University Hospital–Universidad Europea de Madrid), 28046 Madrid, Spain; maria.gaibar@ucjc.edu (M.G.); aponovil@ucm.es (A.N.); diego_malon@yahoo.es (D.M.); beatriz.anton@salud.madrid.org (B.A.-P.); 3 Facultad HM de Ciencias de la Salud, Universidad Camilo José Cela, Instituto de Investigación Sanitaria HMHospitales, 28707 Madrid, Spain; 4 Department of Cell Biology, Faculty of Medicine, Complutense University, 28040 Madrid, Spain; 5 Division of Medical Oncology and Hematology, Department of Medicine, Princess Margaret Cancer Centre and University of Toronto, Toronto, ON M5G 1Z5, Canada; 6 Medical Oncology, Hospital Universitario Ramon y Cajal, 28034 Madrid, Spain; 7 Department of Pathological Anatomy, Hospital Universitario de Getafe, 28905 Madrid, Spain; davidricardo.lujan@salud.madrid.org; 8 Department of Medical Oncology, Hospital Universitario de Getafe, 28905 Madrid, Spain; 9 Department of Medical Oncology, Hospital Universitario de Fuenlabrada, 28942 Madrid, Spain; mmartin.fs@hotmail.com; 10 Department of Pathological Anatomy, Hospital Universitario de Fuenlabrada, 28942 Madrid, Spain; radia.khedaoui@salud.madrid.org; 11 Department of Medical Oncology, Hospital Universitario Fundación Alcorcón, 28922 Madrid, Spain; dmmunoz@salud.madrid.org; 12 Department of Pathological Anatomy, Hospital Universitario Fundación Alcorcón, 28922 Madrid, Spain; fernandojavier.pinedo@salud.madrid.org; 13 Department of Pathological Anatomy, Hospital Universitario de Móstoles, 28935 Madrid, Spain; macarena.boiza@salud.madrid.org (M.B.); p.gomeziglesias@gmail.com (P.G.); 14 Department of Medical Oncology, Hospital Universitario de Móstoles, 28935 Madrid, Spain; silvia.garciaa@salud.madrid.org (S.G.-A.); david.marrupe@gmail.com (D.M.)

**Keywords:** melanoma, immune checkpoints inhibitors, biomarkers, SNPs, toxicity, hepatitis

## Abstract

**Background and Aims:** Immune checkpoint inhibitors (ICIs) have significantly improved survival rates for patients with metastatic melanoma. However, these treatments can lead to immune-related adverse events (irAEs), including hepatitis. This exploratory study sought to identify tumour single-nucleotide polymorphisms (SNPs) associated with the risk of ICI-induced hepatitis in melanoma patients. **Methods:** The study cohort comprised 69 patients with malignant melanoma treated with ICIs at several hospitals in Madrid, Spain. DNA was extracted from formalin-fixed paraffin-embedded tumour biopsies and SNP genotyping was conducted using a MassARRAY platform. **Results:** Significant associations were found between hepatitis risk and 4 of the 20 SNPs examined. A possible risk effect was shown for the variant *GABRP* SNP alleles rs11743438 and rs11743735. Among patients homozygous for these variant alleles (v/v), significantly higher proportions developed hepatitis, 75% and 71.4%, respectively, compared to 32.8% and 21.4, respectively, not developing hepatitis (*p* = 0.046; *p* = 0.013). However, in the same genotype group comparisons, the *RGMA* SNP rs4778080 seemed to have a protective effect, as 100% of patients who developed hepatitis were not in the v/v group for this allele (*p* = 0.043). Additionally, in genotype group comparisons wt/wt versus wt/v + v/v, the *PACRG* SNP rs55733913 was also associated with a higher risk of ICI-induced hepatitis: 66.7% of patients with hepatitis versus 22.8% without hepatitis in genotype group wt/v + v/v (*p* = 0.041). **Conclusions:** This exploratory study identifies candidate tumour SNPs as possible biomarkers to predict the risk of ICI-induced hepatitis, warranting their validation in larger patient cohorts.

## 1. Introduction

Melanoma is globally the ninth most common malignancy and ranks second in skin cancer mortality, with its incidence rising rapidly worldwide, especially among light-skinned populations in Europe and North America [1]. Despite being the least common form of skin cancer, it is the most aggressive and lethal and thus contributes significantly to healthcare costs [2]. Key risk factors include ultraviolet radiation exposure, genetic susceptibility, and environmental changes, with projections predicting a 57–68% increase in cases and related deaths by 2040 [3]. Since 2011, advances in treatment such as immunotherapy and targeted therapies have significantly improved survival in patients with advanced melanoma, and overall survival rates have increased from under 12 months to an undefined, but longer, timeframe for some patients [4].

Immune checkpoints play a pivotal role in regulating normal immune responses and maintaining self-tolerance. The development of monoclonal antibodies that target these checkpoints has revolutionized the treatment of various cancer types including melanoma [5]. Commonly used immune checkpoint inhibitors (ICIs) such as anti-CTLA-4 (cytotoxic T-lymphocyte antigen) and anti-PD-1 (programmed cell death) prevent the immune escape of cancer cells and ultimately cause their death [6]. Ipilimumab, an anti-CTLA-4 monoclonal antibody, was the first ICI to show clinical efficacy in metastatic melanoma alongside other therapies. This led to their FDA approval to treat metastatic disease in 2011 and for their use as adjuvants to other treatments in 2017, laying the groundwork for future immunotherapeutic approaches [7]. The PD-1 inhibitors pembrolizumab and nivolumab, have also improved the treatment landscape for both localized melanoma and extended disease. In effect, in trials like KEYNOTE-006, the efficacy of pembrolizumab over ipilimumab was confirmed, with better progression-free survival and overall survival rates, and 50% of patients still alive after 33 months [8]. When nivolumab was compared to ipilimumab as adjuvant therapy in the CheckMate 238 trial, this agent showed better recurrence-free survival with a lower incidence of severe adverse events, thus solidifying nivolumab’s place for both metastatic melanoma treatment and as adjuvant to melanoma treatment [9]. In the search for synergistic effects of ICIs, combined treatment with ipilimumab and nivolumab resulted in a greater efficacy than single-agent therapies in phase I and II trials, although this combination therapy was associated with a higher rate of severe adverse events compared to monotherapies or treatment discontinuation [10,11]

Immune checkpoints exert their anticancer effects by disrupting the negative interactions that occur between tumour and T-cells, resulting in increased T-cell activity, thereby enhancing the immune system’s actions at the tumour site and inducing on-target off-tumour effects, or immune-related adverse events (irAEs) [12]. The European Society for Medical Oncology (ESMO) and the American Society of Clinical Oncology (ASCO) regularly update and publish guidelines to help healthcare providers manage these irAEs [13]. Toxicity is categorized into five grades based on severity: Grade 1, mild (asymptomatic or mild symptoms); Grade 2, moderate (symptoms that interfere with daily activities); Grade 3, severe (significant disruption of daily life); Grade 4, life-threatening (extreme severity requiring urgent intervention); and Grade 5, death [13]. These guidelines typically recommend suspending ICI treatment, at least temporarily, for any irAEs of grade 2–3 or higher, with consequences including diagnostic interventions, prolonged hospitalization, immunosuppression and a negative impact on the patient’s quality of life.

Hepatotoxicity is a common adverse event of both combined and single-agent ICI therapies, occurring in 25–30% and 2–10% of patients, respectively [14]. ICI-induced hepatitis is diagnosed when levels in blood tests are abnormal (surpassing the upper limit of normal, ULN) of AST (aspartate aminotransferase), ALT (alanine transaminase) and/or bilirubin. Significant elevations in AST and ALT levels are observed in melanoma patients receiving combination treatment based on CTLA-4 and PD-1 inhibition [15]. Additionally, hepatitis ranks as a leading cause of fatal irAEs, accounting for 22% of deaths in patients treated with nivolumab and ipilimumab combined or nivolumab alone, and 16% of deaths in those receiving ipilimumab monotherapy [16]. Some authors have examined the influence of known issues in the development of irAEs, including the specific type and dosage of ICI administered (anti-CTLA-4 or anti-PD-1), different doses, previous viral infections, pre-existing autoimmune conditions, and gut microbiota, among others [17]. Although few studies have addressed biomarkers and ICI toxicity in oncology patients and more specifically in melanoma [18], it seems that patient genetics could play an important role in the management of irAEs. However, previous studies have focused on germline biomarkers as candidate biomarkers of ICI-induced irAEs, yet tumour biomarkers have been underexplored [19,20]. Our hypothesis was that tumour SNPs could predict ICI-induced hepatitis independently of germline genetics. In this context, we here sought to identify clinically useful genetic markers able to predict the development of ICI-induced hepatitis by analyzing twenty single-nucleotide polymorphisms (SNPs) associated with adverse drug reactions in tumour samples from melanoma patients treated with ICIs.

## 2. Materials and Methods

### 2.1. Patients and Samples

This was an observational, multicentre study conducted in patients with malignant melanoma with an indication for treatment with ICIs, and for whom tumour samples were available. An oncologist selected the patients to be enrolled from those receiving care at the University Hospital of Móstoles, University Hospital of the Alcorcón Foundation, University Hospital of Getafe and University Hospital of Fuenlabrada (Madrid, Spain). Inclusion criteria were age greater than or equal to 18 years, melanoma patient receiving treatment with ICIs (monotherapy or combination) between 2010 and 2023 and signed informed consent by the patient for inclusion in the study. Exclusion criteria were other type of malignant tumour, concomitant administration over the previous 4 weeks of another experimental treatment in a clinical trial, and insufficient paraffin-embedded tumour tissue available.

Clinical–pathological variables were collected by medical oncologists in compliance with current regulations on data protection. These included biopsy percentage cellularity, tumour location, sex, treatment, *BRAF* V600E mutation and degree of toxicity associated with the appearance of hepatitis. ICI-induced hepatitis was defined as abnormal blood levels of AST (aspartate aminotransferase), ALT (alanine transaminase), and/or bilirubin referred to the ULN (upper limit of normal). Toxicity was recorded as grade 0 (no toxicity), grade 1 (mild; AST or ALT > ULN to 3.0 × ULN and/or total bilirubin > ULN to 1.5 × ULN), grade 2 (moderate; AST or ALT > 3.0 to ≤ 5 × ULN and/or total bilirubin >1.5 to ≤3 × ULN), grade 3 (severe; AST or ALT 5–20 × ULN and/or bilirubin 3–10 xULN or symptomatic liver dysfunction; fibrosis by biopsy; compensated cirrhosis; and reactivation of chronic hepatitis), grade 4 (life-threatening; AST or ALT > 20 × ULN and/or bilirubin >10 × ULN or decompensated liver function, i.e., ascites, coagulopathy, encephalopathy and coma-), or grade 5 (death due to side effects) [13]. These events were graded according to the Common Terminology Criteria for Adverse Events (CTCAE), version 5.0 [21]. All patients undergoing ICI treatment were routinely subjected to blood tests prior to each treatment cycle. Elevated liver enzyme levels were recorded in real time before treatment administration, and hepatitis was diagnosed at the time of occurrence.

Pathologists from each hospital reviewed the haematoxylin-eosin stained tumour samples choosing the best area to cut sections by macro-dissection to enrich tumour DNA. We only considered samples from biopsies with a percentage cellularity higher than 50%. Total DNA was extracted from 2.5 mm^3^ (5 slices of 5 µm, discarding the 2 first slices) of formalin-fixed paraffin-embedded tumour biopsies with the QIAamp^®^ DNA FFPE Tissue Kit (Qiagen, Germantown, MD, USA), as indicated by the manufacturer. DNA samples were re-suspended in 30 µL of DNAse-free water. The quality and concentration of purified DNA were assessed with the Nanodrop 2000 (Thermo Fisher Scientific, Wilmington, DE, USA). Good-quality DNA samples (A260/A280 ratio of 1.8–2.0) were stored at −20 °C in 96-well plates.

### 2.2. SNP Selection and Genotyping

Twenty SNPs were selected for analysis according to a PubMed (https://pubmed.ncbi.nlm.nih.gov/), accessed on 4 September 2024) relevant literature search using the key words SNP, immunotherapy and cancer. These polymorphisms, chosen because of their interest as germline biomarkers of ICI-induced irAEs [18,19,20], are mainly related to the immune response, regulation of gene expression, central nervous system development, signalling pathways and cell motility (Table 1). The selected 20 SNPs (affecting 14 genes) were *CTLA4* rs11571302, rs1863800, rs3087243, rs4553808, rs16840252, rs231775, rs5742909, *GABRP rs11743438*, rs11743735, *TAGAP-AS1* rs1738074, *LINC00973/RNU6–1263P* rs2062059, *PTPN11* rs2301756, *MIR146A/MIR3142HG* rs2910164, *SEMA5A* rs3026321, *CFAP65/LOC100129175* rs359975, *CFAP36* rs3762513, *RGMA rs4778080*, *PACRG* rs66502444, rs55733913 and *PNPT1* rs782637. Table 1 offers a concise categorization of the genes examined according to their gene ontology-biological process, revealing that the SNPs included in this study are related, either directly or indirectly, to biological processes relevant to immunotherapy responses.

The 20 SNPs were genotyped by the Spanish National Genotyping group (FPGMX-CeGen) using the iPlex^®^Gold chemistry and MassARRAY platform, according to the manufacturer’s instructions (Agena Bioscience, San Diego, CA, USA). The software package Assay Design Suite v3.0 (Agena Bioscience, San Diego, CA, USA) was used to create genotyping assays based on the GRCh38 version. Two separate assays were employed to genotype the 20 SNPs. PCR reactions were conducted in a 5 µL volume, comprising 20 ng of template DNA, 1 × PCR buffer, 2 mM MgCl2, 500 µM dNTPs, and 1 U/reaction of PCR enzyme. PCR reagents were supplied by Agena Bioscience (San Diego, CA, USA). A mixture of PCR primers was prepared with each primer at a final concentration of 100 nM. The thermal cycling protocol included an initial 2 min denaturation at 94 °C, followed by 45 cycles of 94 °C for 30 s, 56 °C for 30 s, and 72 °C for 1 min, concluding with a final 1 min extension at 72 °C. To dephosphorylate unincorporated dTNPs, the PCR products underwent treatment with 0.6 U shrimp alkaline phosphatase (SAP) by incubation at 37 °C for 40 min, followed by enzyme deactivation through heating at 85 °C for 5 min.

The iPLEX Gold assays were conducted in a total volume of 9 µL, comprising 0.222 × iPLEX buffer Plus, 0.5 × iPLEX termination mix, and 0.5 × iPLEX enzyme. An extension primer mix was prepared to achieve a final concentration ranging from 0.73 µM to 1.46 µM for each primer. The thermal cycling protocol began with an initial denaturation at 94 °C for 30 s, followed by 40 cycles at 94 °C for 5 s, incorporating an internal 5-cycle loop at 52 °C for 5 s and 80 °C for 5 s, and concluding with a final extension at 72 °C for 3 min. Subsequently, the iPLEX Gold reaction products were desalted with Clean Resin (Agena Bioscience, San Diego, CA, USA) as per the manufacturer’s instructions. The desalted products were then transferred onto a 384 Spectrochip II using the Chip Prep Module 384 (Agena Bioscience, San Diego, CA, USA). Spectra were obtained through MA4 (Agena Bioscience, San Diego, CA, USA) mass spectrometry, followed by manual examination of the spectra by qualified personnel using MassARRAY Typer software v5.0.9 (Agena Bioscience, San Diego, CA, USA). All experiments were performed in 384-well plates, including negative controls and a set of Coriell samples (Na10861, Na11994 and Na11995) for quality assurance. SNPs with call rates below 95% were excluded to ensure the accuracy and precision of downstream data analyses. The internal controls demonstrated 100% reproducibility and successful genotyping.

### 2.3. Statistical Analysis

The baseline clinical characteristics of all study participants are presented. Means and standard deviations are provided for quantitative variables based on their distribution, as determined by the Kolmogorov–Smirnov normality test. Qualitative variables are expressed as absolute and relative frequencies in percentages. The relationship between different genotypes of each SNP and the occurrence of immunotoxicity-mediated hepatitis was evaluated using contingency tables, either the chi-squared test or Fisher’s exact test, as appropriate. Odds ratios and 95% confidence intervals were calculated for significant associations. Hardy–Weinberg equilibrium was calculated for each gene to confirm the genetic stability of the cohort analyzed. All statistical tests were performed using the package IBM SPSS Statistics for Windows, Version 29.0.2.0 (Armonk, NY, USA). Significance was set at *p* ≤ 0.05.

## 3. Results

The study cohort comprised 69 patients with malignant melanoma who met the inclusion criteria established. Demographic and baseline characteristics are detailed in Table 2. Mean age at the time of diagnosis was 62.1 years (range 28 to 88). Proportions of female/male patients were 53.6%/6.4%. The biological samples analyzed in patients were primary tumours (72.5%), non-nodal metastases (20.3%) and lymph nodes (7.2%) (Table 2). The BRAF mutation V600E was present in 44.1% of all samples. All patients received ICIs: 65.2% nivolumab (*n* = 45), 21.8% pembrolizumab (*n* = 15), 2.9% ipilimumab (*n* = 2) and 10.1% nivolumab + ipilimumab (*n* = 7) (Table 2). ICI-induced hepatitis was confirmed in 11.6% of patients (8 of 69). In seven of these patients, treatment had to be discontinued (toxicity grade 2–3, Table 2).

The genotype frequencies of the 20 SNPs affecting 11 genes are provided in Table 3. Most were within the European frequency range reported in the 1000 Genomes Project Consortium and according to MAF values [22]. The highest wild-type homozygosity rates were observed for the SNPs *CTLA4* rs5742909 (88.4%), *SEMA5A* rs3026321 (85.5%) and *PTPN11* rs2301756 (85.3%) (Table 3). Most patients exhibited carrier status for variant alleles in either homozygous or heterozygous genotypes across multiple SNPs, for instance, *MIR146A/MIR3142HG* rs2910164 (92.4%), *GABRP rs11743438* (84.9%), *PNPT1* rs782637 (83.6%), *CTLA4* rs11571302 (82.4%), *RGMA rs4778080* (82.4%) or *CFAP36* rs3762513 (80.6%) (Table 3). Among these polymorphisms, 2 of them appeared in heterozygosity in roughly half of the subjects: *PNPT1* rs782637 (53.7%) and *CFAP36* rs3762513 (52.2%). The genotype frequencies of the polymorphisms considered here showed good agreement with Hardy–Weinberg equilibrium for 90% of the SNPs except in the case of *CTLA4* rs3087243 and *LINC00973/RNU6–1263P* rs2062059. This indicates the stability of most genes in the population, with no evolution selective forces acting on them.

Hepatitis due to ICI toxicity was recorded in 8 of the 69 patients (11.6%). Treatment was discontinued in 7 of these affected patients in whom ICI toxicity was graded as 2–3, as opposed to grade 1 (Table 2). No relationship was found between the development of hepatitis and the specific ICI therapy received, as patients displaying this adverse event were distributed among all the treatment subgroups (3/8 nivolumab, 1/8 ipilimumab, 1/8 pembrolizumab, 3/8 nivolumab + ipilimumab). Most patients (88.4%) did not develop hepatitis as a consequence of ICI treatment. Initially, comparisons between the two irAE groups (presence/absence of hepatitis) were performed across the three genotypes of each SNP. Table 4 provides genotype frequencies observed in our cohort according to the presence or absence of ICI-induced hepatitis for the 20 SNPs analyzed. For this analysis, we applied Bonferroni correction for multiple comparisons and significance was set at *p* ≤ 0.008; no significant differences were detected (Table 4).

As no differences were noted, we then compared subjects homozygous for the variant allele (v/v) versus the rest of genotypes (wt/wt + wt/v). In this comparison, a possible risk effect of the variant allele was observed for the SNPs rs11743438 and rs11743735 both located on the gene *GABRP*. These two SNPs seemed associated with an increased risk of ICI induced-hepatitis: 6.16-fold (95% CI: 1.13–33.43) in the case of rs11743438 and 9.17-fold (95% CI: 1.58–53.27) for rs11743735 (Table 5). Accordingly, we observed a significantly higher number of patients homozygous (v/v) for the *GABRP* SNPs rs11743438 and rs11743735 in the hepatitis group, 75% and 71.4%, respectively, compared to the non-hepatitis group, 32.8% and 21.4, respectively (*p* = 0.046; *p* = 0.013, Table 5). However, in the same comparison, the *RGMA* SNP rs4778080 appeared to have a protective effect, as 100% of patients who developed hepatitis were not in the v/v group. For this SNP, 100% of patients developing hepatitis had genotypes wt/wt or wt/v versus 60% of patients without this irAE (*p* = 0.043, Table 5).

We also examined whether carrying a single copy of the variant allele could lead to an increased risk of developing immune-induced hepatitis. This was an effect observed for the *PACRG* SNP rs55733913 given the presence of 66.7% of subjects with ICI-induced hepatitis in the group of patients with 1 or 2 C alleles (v/v + wt/v) versus 22.8% of patients without hepatitis in this same group (*p* = 0.041, Table 5). In this case, the risk of developing ICI induced-hepatitis was 6.77-fold (95% CI: 1.11–41.22) in patients with one or two copies of the variant allele (Table 5).

## 4. Discussion

This exploratory study examines the development of hepatitis as an adverse effect of ICI treatment in 69 patients with melanoma. The patients’ tumours were genotyped according to the presence or absence of 20 SNPs known for their role as germline genetic determinants of irAEs, but whose role in tumour genetics is currently unclear [18,19,20]. These polymorphisms are involved in processes such as immunologic responses, central nervous system development, post-transcriptional regulation of gene expression and signalling pathways, among others (see Table 1). Our results indicate a range of allele frequencies of the 20 SNPs examined in our cohort within that reported for other European populations [22]. Overall, these frequencies were in good agreement with Hardy–Weinberg equilibrium, with two exceptions, that of the variants *CTLA4* rs3087243 and *LINC00973/RNU6–1263P* rs2062059. A possible explanation for this discrepancy could be an association between the presence of some alleles and an increased or decreased risk of several types of cancer such that the frequency of a variant allele in a cohort of oncology patients could differ from its frequency in a sample of healthy individuals within the 1000 Genomes database. For instance, Yang et al. found a relationship between the rs3087243 polymorphism of the *CTLA4* gene and susceptibility to hepatocellular carcinoma [23], which is in line with the higher frequency of the variant allele observed in our melanoma patients than that reported for healthy populations [22]. To our knowledge, there are no literature reports of the allele frequencies of *LINC00973/RNU6–1263P* rs2062059.

While the prognosis of advanced malignant melanoma has been historically poor, with overall survival typically less than a year [14], the advent of immune checkpoint inhibitors has significantly improved five-year relative survival rates for patients with metastatic melanoma [24]. This progress, however, comes with the drawback of severe adverse effects in some individuals. In a comprehensive systematic review of 13 studies published from 2010 to 2021 examining the risk of serious side effects associated with immune checkpoint blockade [14], a key finding was the notably lower incidence of severe irAEs associated with PD-1 inhibition monotherapy (5.1%) compared to combination therapy (23.2%). However, while the combined regimen of nivolumab and ipilimumab was found to carry the highest risk of irAEs, it also provided the most favourable overall survival outcomes. After five years, 52% of patients receiving this combination therapy were still alive, surpassing the survival rates of both nivolumab monotherapy (44%) and ipilimumab monotherapy (26%) [25].

Hepatitis is a common side effect of both combination and single-agent ICI therapy and occurs in 25–30% and 2–10% of patients, respectively [13]. In our cohort of patients, the combination therapy group showed the highest hepatitis incidence at 42.8% (3 out of 7 patients), while in the anti-PD1 monotherapy group, 6.7% of nivolumab-treated patients (3/45) and 6.7% of pembrolizumab-treated patients (1/15) developed hepatitis. As described by other authors, hepatitis is also a significant cause of fatal irAEs and accounted for 22% of deaths in patients receiving nivolumab and ipilimumab combined or nivolumab monotherapy, and 16% of deaths in those treated with ipilimumab alone [16].

Studies addressing the use of SNPs as markers of ICI-induced toxicity in patients with melanoma have been limited and yielded inconsistent results. For instance, de Joode et al. correlated several *CTLA4* SNPs with adverse effects. Specifically, rs3087243 was associated with an increased risk of auto- and alloimmunity in a germline genetics study in patients with advanced stage melanoma [26]. Another investigation revealed that *CTLA4* rs4553808 was associated with an increased risk of autoimmune disease including endocrinopathy in melanoma patients treated with ipilimumab [27]. Data such as these could be useful to select the best anti-CTLA4 ICI treatment option and dose. In our study, only 9 out of 69 patients (13%) received ipilimumab treatment, either as a monotherapy or in combination with another ICI. We were unable to obtain significant risk associations for any of the seven *CTLA4* SNPs, including the previously mentioned rs3087243 and rs4553808. However, among our tumour samples we did identify three SNPs associated with an increased risk of ICI-induced hepatitis. Two of these were polymorphisms of the *GABRP* gene (GABA A receptor subunit pi), which encodes a multisubunit chloride channel mediating the fastest inhibitory synaptic transmission pathway in the central nervous system. A possible risk effect was shown for both *GABRP* SNPs rs11743438 and rs11743735: a significantly higher rate of homozygous patients for the variant allele (v/v) was recorded among the patients with hepatitis, 75% and 71.4%, respectively, than among those without hepatitis, 32.8% and 21.4, respectively (*p* = 0.046; *p* = 0.013, Table 5). Similar results were obtained by Abdel-Wahad et al. in the first exploratory GWAS performed to identify germline genetic variants associated with the risk of developing irAEs in melanoma patients treated with ICIs [18]. These last authors also described a link between the presence of *GABRP rs11743438* and rs11743735 and a more likely risk of developing irAEs (OR = 4.3, 95% CI 2.3–8.0, *p* = 5.56 × 10^–6^ and OR = 4.3, 95% CI 2.3–8.8, *p* = 8.34 × 10^–6^, respectively). We observed a similar effect of the SNP rs55733913 of the *PACRG* gene (parkin co-regulated gene) associated with chaperones and the suppression of unfolding processes in the nervous system. The association of this SNP with ICI-induced hepatitis was here observed with only one dose of the variant allele (wt/v + v/v versus wt/wt) in that the rate of genotypes with one or two variant alleles (wt/v + v/v) was significantly higher in the group of patients with hepatitis (66.7%) than the group of patients without hepatitis (22.8%) (*p* = 0.041, Table 5). This observation is inconsistent with the findings of Abdel-Wahad, who reported a decreased risk of developing irAEs associated with *PACRG rs55733913* in germline melanoma patient samples [18]. Others have also identified specific alleles associated with certain types of irAEs, for instance, those of HLA genes: DR4 linked to diabetes and hepatitis, and DR8 to hypothyroidism [28]. HLA-A class I homozygosity has been significantly linked to the occurrence of some irAEs such as colitis and hepatitis in melanoma patients [29]. These findings suggest that HLA genotyping, besides SNP analysis, could be a predictive tool to identify patients at risk of specific irAEs prior to ICI therapy onset in patients with melanoma.

Our results also point to a possible protective effect of the rs4778080 variant of the gene *RGMA* (repulsive guidance molecule BMP co-receptor A), related to central nervous system development. Thus, 100% of our patients with hepatitis were not in the v/v group. In fact, 100% of patients developing hepatitis showed the genotypes wt/wt or wt/v versus 60% of patients without hepatitis carrying the same genotypes (*p* = 0.043, Table 5). Abdel-Wahab et al. also associated lower odds of developing irAEs with the detection of this *RGMA* SNP rs4778080 [18] in blood samples from melanoma patients. Other authors have also linked germline SNPs, such as *PDCD1* rs2227981 with a lower risk of treatment-related toxicity of any grade in non-small cell lung cancer patients receiving anti-PD1 nivolumab treatment [30]. Recently, de Joode et al. [26] proposed two *CTLA-4* SNPs as predictors of treatment outcome in melanoma patients undergoing ipilimumab therapy. The wt genotypes of the *CTLA-4* gene SNPs Jo27T > C and Jo31G > T were linked to overall survival. The present exploratory study presents the first results obtained in tumour specimens as opposed to blood samples. We propose that the genetic profiles of tumours with such high heterogeneity of mutations could provide excellent information for both selecting the most suitable treatment and to avoid possible irAEs in patients. However, the utility of tumour genetics and not only the genetic germline of patients for the management of ICI treatment toxicity requires confirmation in a prospective large trial. Besides genetic factors, the relationship between hepatitis risk and ICI or other anti-tumour therapies is becoming increasingly important. A recent study has shown that ICI and targeted therapy combined may be correlated with a higher risk of hepatitis compared with ICI alone, while ICI plus chemotherapy was also related to a risk of hepatitis comparable to that related to ICI alone in patients with non-small-cell lung cancer [31]. As other authors have mentioned, several challenges need to be addressed in the management of melanoma patients including handling toxicity, evaluating expenses, and finding predictive biomarkers to assist in patient selection for therapy [32].

Despite its limited sample size, this exploratory study offers interesting preliminary data that provide direction for more extensive research focused on improving our understanding of the tumour genetics underlying ICI-induced hepatitis in melanoma patients. This could be useful to identify the optimal treatment that will offer the best outcome while inducing the least toxicity. As far as we know, our data are the first derived specifically from melanoma tumour specimens.

## 5. Conclusions

In this retrospective exploratory study, we analyzed 20 SNPs of different genes to assess their potential role as biomarkers of the clinical risk of developing ICI-induced hepatitis in a cohort of patients with melanoma treated with nivolumab, pembrolizumab, ipilimumab, and ipilimumab + nivolumab. Four interesting SNPs possibly associated with ICI-induced hepatitis were identified in our tumour specimens: three of them, *GABRP rs11743438, GABRP* rs11743735, and *PACRG rs55733913* were found linked to a higher risk of hepatitis, while *RGMA rs4778080* seemed to protect against this adverse event. Our findings require confirmation in larger cohorts of patients with melanoma. As ICIs are becoming more and more frequently used to treat many different cancer types, we predict that tumour SNPs could serve as useful toxicity biomarkers to guide treatment selection while avoiding adverse effects such as hepatitis.

## Figures and Tables

**Table 1:** The twenty SNPs selected, and classification of the genes affected by them according to the biological process they are involved in (gene ontology). (wt = wild-type allele, v = variant allele).

Gene Ontology	Gene	SNP wt > v
Immunologic response	*CTLA4* (cytotoxic T-lymphocyte-associated protein 4)	rs11571302 G > T
		rs1863800 C > T
		rs3087243 G > A
		rs4553808 A > G
		rs16840252 C > T
		rs231775 A > G
		rs5742909 C > T
	*MIR146A/* *MIR3142HG*	rs2910164 C > G
Central nervous system development	*GABRP* (gamma-aminobutyric acid type A receptor subunit pi)	rs11743438 C > T
		rs11743735 C > A
	*SEMA5A* (semaphorin 5A)	rs3026321 A > T
	*RGMA* (repulsive guidance molecule BMP co-receptor A)	rs4778080 G > T
Post-transcriptional regulation of gene expression	*TAGAP-AS1* (antisense RNA 1, lncRNA)	rs1738074 T > C
	*PNPT1* (polyribonucleotide nucleotidyltransferase 1)	rs782637 T > C
	*LINC00973/RNU6–1263P* (long intergenic non-protein coding RNA 973, U6 small nuclear 1263, pseudogene)	rs2062059 A > G
Signalling pathways	*PTPN11* (protein tyrosine phosphatase non-receptor type 11)	rs2301756 A > G
Cell motility	*CFAP65/* *LOC100129175* (cilia- and flagella-associated protein 65, lncRNA)	rs359975 C > G
	*CFAP36* (cilia- and flagella-associated protein)	rs3762513 C > T
Chaperons and chaperonins	*PACRG* (parkin co-regulated gene protein)	rs55733913 T > C
		rs66502444 A > T

**Table 2:** Patient demographic and baseline characteristics (*n* = 69).

Patient Characteristics		No. (%)
Sex	Male	32 (46.4%)
	Female	37 (53.6%)
Biopsy specimen	Primary tumour	50 (72.5%)
	Non-nodal metastasis	14 (20.3%)
	Lymph nodes	5 (7.2%)
BRAF status	Wild type	38 (55.9%)
	V600E mutation	30 (44.1%)
Immunotherapy regimen	Nivolumab	45 (65.2%)
	Pembrolizumab	15 (21.8%)
	Ipilimumab	2 (2.9%)
	Ipilimumab + nivolumab	7 (10.1%)
Hepatitis toxicity	Grade 0	61 (88.4%)
	Grade1	1 (1.4%)
	Grade 2	3 (4.4%)
	Grade 3	4 (5.8%)
	Grade 4–5	0 (0%)

**Table 3:** Genotype and allele frequencies of the 20 SNPs examined. European frequencies from the 1000 Genomes database are indicated [22]. (wt = wild-type allele, v = variant allele). MAF: minor allele frequency observed in 1000 Genomes Phase 3 combined population. * MAF from gnomAD exomes v4.1 (10).

SNP wt > v	Genotype Frequency	Allele Frequency	1000 Genomes European Allele Frequency	MAF
rs11571302 G > T	GG = 12 (17.6%) GT = 33 (48.5%) TT = 23 (33.9%)	G = 41.9% T = 58.1%	G = 51.1% T = 48.9%	0.43 (T)
rs1863800 C > T	CC = 16 (23.9%) CT = 33 (49.2%) TT = 18 (26.9%)	C = 48.5% T = 51.5%	C = 55.0% T = 45.0%	0.41 (T)
rs3087243 G > A	GG = 14 (23.7%) GA = 10 (16.9%) AA = 35 (59.4%)	G = 32.2% A = 67.8%	G = 53% A = 47%	0.37 (A)
rs4553808 A > G	AA = 41 (63.1%) AG = 23 (35.4%) GG = 1 (1.5%)	A = 80.8% G = 19.2%	A = 83.1% G = 16.9%	0.13 (G)
rs16840252 C > T	CC = 42 (61.8%) CT = 25 (36.8%) TT = 1 (1.4%)	C = 80.1% T = 19.9%	C = 83.1% T = 16.9%	0.13 (T)
rs231775 A > G	AA = 37 (54.4%) AG = 25 (36.8%) GG = 6 (8.8%)	A = 72.8% G = 27.2%	A = 64.1% G = 35.9%	0.43 (G)
rs5742909 C > T	CC = 61 (88.4%) CT = 8 (11.6%) TT = 0	C = 94.2% T = 5.8%	C = 91.6% T = 8.4%	0.05 (T)
rs2910164 C > G	CC = 5 (7.6%) CG = 22 (33.3%) GG = 39 (59.1%)	C = 24.3% G = 75.7%	C = 26% G = 74%	0.26 (C)
rs11743438 C > T	CC = 10 (15.1%) CT = 31 (47.0%) TT = 25 (37.9%)	C = 38.6% T = 61.4%	C = 45.9% T = 54.1%	0.46 (T)
rs11743735 C > A	CC = 20 (31.7%) CA = 26 (41.3%) AA = 17 (27.0%)	C = 52.4% A = 47.6%	C = 61.2% A = 38.8%	0.31 (A)
rs3026321 A > T	AA = 59 (85.5%) AT = 10 (14.5%) TT = 0	A = 92.8% T = 7.2%	A = 91.4% T = 8.6%	0.05 (T)
rs4778080 G > T	GG = 12 (17.6%) GT = 32 (47.1%) TT = 24 (35.3%)	G = 41.2% T = 58.8%	G = 36.5% T = 63.5%	0.32 (G)
rs1738074 T > C	TT = 17 (25.8%) TC = 32 (48.4%) CC = 17 (25.8%)	T = 50% C = 50%	T = 43.3% C = 56.7%	0.47 (C)
rs782637 T > C	TT = 11 (16.4%) TC = 36 (53.7%) CC = 20 (29.9%)	T = 43.3% C = 56.7%	T = 46.7% C = 53.3%	0.33 (C)
rs2062059 A > G	AA = 32 (66.6%) AG = 9 (18.8%) GG = 7 (14.6%)	A = 76% G = 24%	A = 62.1% G = 37.9%	0.32 (G)
rs2301756 A > G	AA = 58 (85.3%) AG = 10 (14.7%) GG = 0	A = 92.6% G = 7.4%	A = 90.3% G = 9.7%	0.37 (G)
rs359975 C > G	CC = 53 (79.1%) CG = 12 (17.9%) GG = 2 (3.0%)	C = 88.1% G = 11.9%	C = 87.7% G = 12.3%	0.26 * (G)
rs3762513 C > T	CC = 13 (19.4%) CT = 35 (52.2%) TT = 19 (28.4%)	C = 45.5% T = 54.5%	C = 47.8% T = 52.2%	0.31 (T)
rs55733913 T > C	TT = 46 (73.0%) TC =15 (23.8%) CC = 2 (3.2%)	T = 84.9% C = 15.1%	T = 76.7% C = 23.3%	0.20 (C)
rs66502444 A > T	AA = 52 (78.8%) AT = 13 (19.7%) TT = 1 (1.5%)	A = 88.7% T = 11.3%	A = 81.7% T = 18.3%	0.13 (T)

**Table 4:** Patients with ICI-induced hepatitis by genotype for the 20 SNPs analyzed. Sample sizes (*n*) and percentages are detailed by SNP genotype.

SNP	ICI-Induced Hepatitis *n* (%)	No ICI-Induced Hepatitis *n* (%)	TOTAL *n* (%)	*p*-Value
rs11571302	GG = 2 (25%) GT = 4 (50%) TT = 2 (25%)	GG = 10 (16.7%) GT = 29 (48.3%) TT = 21 (35%)	GG = 12 (17.6%) GT = 33 (48.5%) TT = 23 (33.9%)	0.78
rs1863800	CC = 2 (25%) CT = 5 (62.5%) TT = 1 (12.5%)	CC = 14 (23.7%) CT = 28 (47.5%) TT = 17 (28.8%)	CC = 16 (23.9%) CT = 33 (49.2%) TT = 18 (26.9%)	0.60
rs3087243	GG = 2 (25%) GA = 1 (12.5) AA = 5 (62.5%)	GG = 12 (23.5%) GA = 9 (17.6%) AA = 30 (58.9%)	GG = 14 (23.7%) GA = 10 (16.9%) AA = 35 (59.4%)	0.94
rs4553808	AA = 4 (57.1%) AG = 3 (42.8%) GG = 0	AA = 37 (63.8%) AG = 20 (34.5%) GG = 1 (1.7%)	AA = 41 (63.1%) AG = 23 (35.4%) GG = 1 (1.5%)	0.87
rs16840252	CC = 4 (50%) CT = 4 (50%) TT = 0	CC = 38 (63.3%) CT = 21 (35%) TT = 1 (1.7%)	CC = 42 (61.8%) CT = 25 (36.8%) TT = 1 (1.4%)	0.68
rs231775	AA = 5 (62.5%) AG = 2 (25%) GG = 1 (12.5%)	AA = 32 (53.3%) AG = 23 (38.4%) GG = 5 (8.3%)	AA = 37 (54.4%) AG = 25 (36.8%) GG = 6 (8.8%)	0.74
rs5742909	CC = 7 (87.5%) CT = 1 (12.5%) TT = 0	CC = 54 (88.5%) CT = 7 (11.5%) TT = 0	CC = 61 (88.4%) CT = 8 (11.6%) TT = 0	1.00
rs2910164	CC = 0 CG = 1 (14.3%) GG = 6 (85.7%)	CC = 5 (8.5%) CG = 21 (35.6%) GG = 33 (55.9%)	CC = 5 (7.6%) CG = 22 (33.3%) GG = 39 (59.1%)	0.30
rs11743438	CC = 0 CT = 2 (25%) TT = 6 (75%)	CC = 10 (17.2%) CT = 29 (50.0%) TT = 19 (32.8%)	CC = 10 (15.1%) CT = 31 (47.0%) TT = 25 (37.9%)	0.06
rs11743735	CC = 1 (14.3%) CA = 1 (14.3%) AA = 5 (71.4%)	CC = 19 (33.9%) CA = 25 (44.6%) AA = 12 (21.4%)	CC = 20 (31.7%) CA = 26 (41.3%) AA = 17 (27.0%)	0.02
rs3026321	AA = 8 (100%) AT = 0 TT = 0	AA = 51 (83.6%) AT = 10 (16.4%) TT = 0	AA = 59 (85.5%) AT = 10 (14.5%) TT = 0	0.59
rs4778080	GG = 2 (25%) GT = 6 (75%) TT = 0	GG = 10 (16.7%) GT = 26 (43.3%) TT = 24 (40%)	GG = 12 (17.6%) GT = 32 (47.1%) TT = 24 (35.3%)	0.08
rs1738074	TT = 3 (37.5%) TC = 3 (37.5%) CC = 2 (25%)	TT = 14 (24.1%) TC = 29 (50%) CC = 15 (25.9%)	TT = 17 (25.8%) TC = 32 (48.4%) CC = 17 (25.8%)	0.70
rs782637	TT = 2 (28.6%) TC = 3 (42.8%) CC = 2 (28.6%)	TT = 9 (15%) TC = 33 (55%) CC = 18 (30%)	TT = 11 (16.4%) TC = 36 (53.7%) CC = 20 (29.9%)	0.64
rs2062059	AA = 2 (66.7%) AG = 1 (33.3%) GG = 0	AA = 30 (66.7%) AG = 8 (17.8%) GG = 7 (15.6%)	AA = 32 (66.6%) AG = 9 (18.8%) GG = 7 (14.6%)	0.66
rs2301756	AA = 6 (85.7%) AG = 1 (14.3%) GG = 0	AA = 52 (85.2%) AG = 9 (14.8%) GG = 0	AA = 58 (85.3%) AG = 10 (14.7%) GG = 0	1.00
rs359975	CC = 7 (100%) CG = 0 GG = 0	CC = 46 (76.7%) CG = 12 (20%) GG = 2 (3.3%)	CC = 53 (79.1%) CG = 12 (17.9%) GG = 2 (3.0%)	0.36
rs3762513	CC = 3 (37.5%) CT = 3 (37.5%) TT = 2 (25%)	CC = 10 (16.9%) CT = 32 (54.2%) TT = 17 (28.9%)	CC = 13 (19.4%) CT = 35 (52.2%) TT = 19 (28.4%)	0.38
rs55733913	TT = 2 (33.3%) TC = 3 (50%) CC = 1 (16.7%)	TT = 44 (77.2%) TC = 12 (21.1%) CC = 1 (1.7%)	TT = 46 (73.0%) TC =15 (23.8%) CC = 2 (3.2%)	0.03
rs66502444	AA = 3 (50%) AT = 3 (50%) TT = 0	AA = 49 (81.7%) AT = 10 (16.7%) TT = 1 (1.6%)	AA = 52 (78.8%) AT = 13 (19.7%) TT = 1 (1.5%)	0.14

**Table 5:** Genotype groups compared according to the presence or absence of ICI-induced hepatitis. Only SNPs yielding significant results are detailed. Odds ratios and 95% confidence intervals are shown.

SNP (Gene)	Genotype Groups Compared	Hepatitis *n* (%)		OR (95% CI)	*p*-Value
		Yes	No		
rs11743438 *(GABRP)*	v/v	6 (75%)	19 (32.8%)	6.16 (1.13–33.43)	0.046
	wt/wt + wt/v	2 (25%)	39 (67.2%)		
rs11743735 *(GABRP)*	v/v	5 (71.4%)	12 (21.4%)	9.17 (1.58–53.27)	0.013
	wt/wt + wt/v	2 (28.6%)	44 (78.6%)		
rs4778080 *(RGMA)*	v/v	0	24 (40%)	-	0.043
	wt/wt + wt/v	8 (100%)	36 (60%)		
rs55733913 (*PACRG)*	v/v + wt/v wt/wt	4 (66.7%) 2 (33.3%)	13 (22.8%) 44 (77.2%)	6.77 (1.11–41.22)	0.041

## Data Availability

Data is contained within the article. The original contributions presented in this study are included in the article. Further inquiries can be directed to the corresponding author(s).

## Abbreviations

The following abbreviations are used in this manuscript:

ICI	Immune checkpoint inhibitor
irAE	Immune-related adverse event
SNP	Single-nucleotide polymorphisms
CTLA-4	Cytotoxic T-lymphocyte antigen
PD-1	Programmed cell death
wt	Wild-type allele
v	Variant allele
